# 2-{(*E*)-*N*-[2-(1*H*-Inden-3-yl)eth­yl]imino­meth­yl}-1*H*-imidazole

**DOI:** 10.1107/S1600536811013286

**Published:** 2011-04-16

**Authors:** Zhao Li, Chong Tian, Wanli Nie, Maxim V. Borzov

**Affiliations:** a Key Laboratory of Synthetic and Natural Chemistry of the Ministry of Education, College of Chemistry and Material Science, the North-West University of Xi’an, Taibai Bei avenue 229, Xi’an 710069, Shaanxi Province, People’s Republic of China

## Abstract

The asymmetric unit of the title compound, C_15_H_15_N_3_, contains two crystallographically independent mol­ecules with very similar geometries. The imidazole and indenyl planes are approximately orthogonal, making dihedral angles of 88.21 (9) and 83.08 (9)%deg; in the two independent molecules. In the crystal, the imidazole units are linked by N—H⋯N hydrogen bonds into chains parallel to the 101) plane stretched in the diagonal direction [translation vector (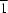,1,0); *C*(4) motif]. Within a chain, there are two types of symmetrically non-equivalent alternating H-bonds which slightly differ in their parameters.

## Related literature

For the structural parameters of 3-organyl substituted 1*H*-indenes (organic structures only), see: Sun *et al.* (2010 ▶) and references cited therein. For the structural parameters of 2-organyl-1*H*-imidazoles (organic structures only, not bi- or oligocyclic, non-ionic, recent publications only), see: Lassalle-Kaiser *et al.* (2006 ▶). For the structural parameters of Li, Ti, and Zr complexes derived from 1*H*-imidazol(in)-2-yl side-chain-functionalized cyclo­penta­dienes see: Krut’ko *et al.* (2006 ▶); Nie *et al.* (2008 ▶); Wang *et al.* (2009 ▶); Ge *et al.* (2010 ▶). For the structural parameters of 1*H*-imidazol(in)-2-yl side-chain-functionalized 3-substituted 1*H*-indene and Li-indenide, see: Sun *et al.* (2009 ▶, 2010 ▶). For graph-set notation, see: Etter *et al.* (1990 ▶); Bernstein *et al.* (1995 ▶). For a description of the Cambridge Structural Database, see: Allen (2002 ▶). For preparation of 2-(1*H*-inden-3-yl)ethanamine, see: Winter *et al.* (1967 ▶).
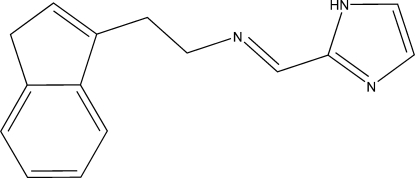


## Experimental

### 

#### Crystal data

C_15_H_15_N_3_
*M* *_r_* = 237.30
Orthorhombic, 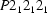

*a* = 5.8827 (5) Å
*b* = 8.3326 (7) Å
*c* = 51.909 (4) Å
*V* = 2544.5 (4) Å^3^
*Z* = 8
Mo *K*α radiation
μ = 0.08 mm^−1^
*T* = 296 K
0.36 × 0.22 × 0.14 mm

#### Data collection

Bruker SMART APEXII diffractometer
Absorption correction: multi-scan (*SADABS*; Sheldrick, 1996 ▶) *T*_min_ = 0.973, *T*_max_ = 0.990
13315 measured reflections
2939 independent reflections
2328 reflections with *I* > 2σ(*I*)
*R*_int_ = 0.041

#### Refinement

*R*[*F* ^2^ > 2σ(*F* ^2^)] = 0.044
*wR*(*F* ^2^) = 0.113
*S* = 1.03
2939 reflections
333 parameters
H atoms treated by a mixture of independent and constrained refinement
Δρ_max_ = 0.14 e Å^−3^
Δρ_min_ = −0.21 e Å^−3^

### 

Data collection: *APEX2* (Bruker, 2007 ▶); cell refinement: *SAINT* (Bruker, 2007 ▶); data reduction: *SAINT*; program(s) used to solve structure: *SHELXS97* (Sheldrick, 2008 ▶); program(s) used to refine structure: *SHELXL97* (Sheldrick, 2008 ▶); molecular graphics: *SHELXTL* (Sheldrick, 2008 ▶) and *OLEX2* (Dolomanov *et al.*, 2009 ▶); software used to prepare material for publication: *SHELXTL* and *OLEX2*.

## Supplementary Material

Crystal structure: contains datablocks I, global. DOI: 10.1107/S1600536811013286/im2276sup1.cif

Structure factors: contains datablocks I. DOI: 10.1107/S1600536811013286/im2276Isup2.hkl

Additional supplementary materials:  crystallographic information; 3D view; checkCIF report

## Figures and Tables

**Table 1:** Hydrogen-bond geometry (Å, °)

*D*—H⋯*A*	*D*—H	H⋯*A*	*D*⋯*A*	*D*—H⋯*A*
N1*A*—H1*A*⋯N2*B*	0.80 (4)	2.16 (4)	2.935 (4)	162 (4)
N1*B*—H1*B*⋯N2*A*^i^	0.92 (4)	2.10 (4)	3.006 (4)	170 (3)

Symmetry code: (i) 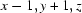.

**Fig. 1.**
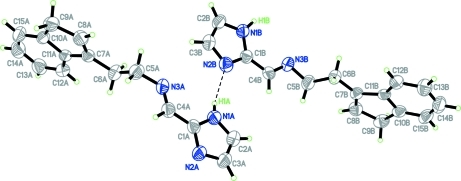
Asymmetric unit of the compound I with labelling and thermal ellipsoids at the 50% probability level. Hydrogen bond is depicted as a dashed line.

**Fig. 2.**
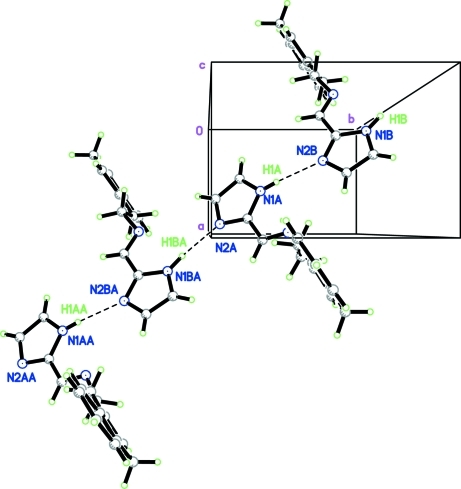
Chain-assembling of the molecules of I. Prospective view along c-axis. Only atoms participating in the hydrogen bond formation are labeled. Hydrogen bonds are depicted as dashed lines.

## supplementary crystallographic information

### Comment

1*H*-Imidazol(in)-2-yl side-chain functionalized cyclopentadiene-type (Cp) ligands were introduced into the organometallic chemistry, and, particularly into that of the Group 4 transition metals, not long ago (Krut'ko *et al.*, 2006; Nie *et al.*, 2008; Wang *et al.*, 2009; Sun *et al.*, 2009; Sun *et al.*, 2010; Ge *et al.*, 2010). All these compounds are usually considered to be prospective precursors for catalytic systems capable to effectively polymerize ethylene and α-olefins. However, in all of these previously reported ligands, the Cp- and imidazol-2-yl groups are linked by a C_1_– or C_2_-hydrocarbon bridge. Incorporating into the bridge another heteroatom groups capable of coordination towards a metal centre presents, this way, a logical step forward in the ligand design development. This contribution reports the first structural characterization of a potent tridentate ligand of the type where Cp- (1*H*-inden-3-yl) and 1*H*-imidazol-2-yl groups are connected with a bridge with a C═N imino-function.

The achiral title compound, C_15_H_15_N_3_, I, was prepared by a condensation reaction of 2-(1*H*-inden-3-yl)ethanamine and 1*H*-imidazol-2-carbaldehyde. It crystallizes in a chiral space group *P*2_1_2_1_2_1_, with the *c*-axis of the lattice being very long comparatively to the others [51.909 (4) Å]. The asymmetric unit of I is presented by two crystallographically independent molecules with very close geometries (see Fig. 1). Imidazole moieties of the asymmetric unit are linked by NH···N hydrogen bonds and the units assemble in chains parallel to *a*0*b* plane stretched in the diagonal direction [translation vector (–1,1,0); *C*(4) motif; see Fig. 2]. Within a chain, these hydrogen bonds slightly alternate (see Table).

Both indenyl groups are planar within 0.03 Å and nearly parallel one to each other [interplane angle 1.44 (6)°]. Within the independent molecules, the imidazole and indenyl r. m. s. planes are approximately orthogonal [interplane angles 88.21 (9) and 96.92 (9)°]. However, the imidazole rings in the units form a noticible interplane angle [7.43 (11)°] what could be a result of their mutual hydrogen binding. The same binding could also be a reason of noticible twisting of the C═N fragments in respect to the imidazole ring planes [torsion angles 7.5 (4) and 7.3 (4)°].

Analysis of the Cambridge Structural database [CSD; Version 5.27, release May 2009; Allen, 2002; 317 entries, 483 fragments] reveals that the observed C═ N distances in I [1.251 (4) and 1.253 (4) Å] are close to the median value for C═N bond in Schiff bases derived from primary aliphatic amines and aromatic (and/or heteroaromatic) aldehydes (1.27 Å). As for the 1*H*-inden-3-yl and 1*H*-imidazol-2-yl groups, all the bond lengths and angles are within normal ranges (for references, see Related literature section).

### Experimental

Methanol was refluxed with Mg powder until the metal dissolved and then distilled from over Mg(OMe)_2_. 1*H*-Imidazol-2-carbaldehyde was purchased from *Fluka*. 2-(1*H*-inden-3-yl)ethanamine was prepared as described by Winter *et al.*, 1967.

Compound I: Solutions of 2-(1*H*-inden-3-yl)ethanamine (1.56 g, 10 mmol) and 1*H*-imidazol-2-carbaldehyde (0.96 g, 10 mmol) in anhydrous methanol (total amount 20 ml) were mixed under stirring at 253 K, the reaction mixture was kept at this temperature for 6 h and then cooled down to 233 K. The solution was removed from the wthite thin-crystalline precipitate with a canula. The precipitate was washed with small portions of cold diethyl ether and dried on the high-vacuum line what gave 1.85 g (78%) of I. Single crystal of I suitable for the X-ray diffraction analysis was prepared by re-crystallization from anhydrous methanol (slow evaporation, ambient temperature).

### Refinement

Non-H atoms were refined anisotropically. All H atoms except of the ones located at nitrogen atom of the imidazole groups were treated as riding atoms with distances C—H = 0.97 (CH_2_), 0.93 Å (C_Ar_H), and *U*_iso_(H) = 1.2 *U*_eq_(C), and 1.2 *U*_eq_(C), respectively. H atoms at N atoms were found from the difference Fourier synthesis and refined isotropically. Despite of the fact that an achiral compound I crystallizes in a chiral space group *P*2_1_2_1_2_1_, neither the absolute structure determination nor approval of the inversion twinning was possible due to evident reasons (Mo-*K*α radiation with no atoms heavier than nitrogen). Thus, the refinement for I was preformed with the Friedel opposites merged (MERG 3 instruction).

### Crystal data

**Table:**

C_15_H_15_N_3_	*F*(000) = 1008
*M**_r_* = 237.30	*D*_x_ = 1.239 Mg m^−^^3^
Orthorhombic, *P*2_1_2_1_2_1_	Mo *K*α radiation, λ = 0.71073 Å
Hall symbol: P 2ac 2ab	Cell parameters from 8457 reflections
*a* = 5.8827 (5) Å	θ = 2.4–28.2°
*b* = 8.3326 (7) Å	µ = 0.08 mm^−^^1^
*c* = 51.909 (4) Å	*T* = 296 K
*V* = 2544.5 (4) Å^3^	Block, colourless
*Z* = 8	0.36 × 0.22 × 0.14 mm

### Data collection

**Table:**

Bruker SMART APEXII diffractometer	2939 independent reflections
Radiation source: fine-focus sealed tube	2328 reflections with *I* > 2σ(*I*)
graphite	*R*_int_ = 0.041
Detector resolution: 8.333 pixels mm^-1^	θ_max_ = 26.0°, θ_min_ = 2.4°
φ and ω scans	*h* = −7→5
Absorption correction: multi-scan (*SADABS*; Sheldrick, 1996)	*k* = −10→10
*T*_min_ = 0.973, *T*_max_ = 0.990	*l* = −64→61
13315 measured reflections	

### Refinement

**Table:**

Refinement on *F*^2^	Primary atom site location: structure-invariant direct methods
Least-squares matrix: full	Secondary atom site location: difference Fourier map
*R*[*F*^2^ > 2σ(*F*^2^)] = 0.044	Hydrogen site location: inferred from neighbouring sites
*wR*(*F*^2^) = 0.113	H atoms treated by a mixture of independent and constrained refinement
*S* = 1.03	*w* = 1/[σ^2^(*F*_o_^2^) + (0.057*P*)^2^ + 0.4576*P*] where *P* = (*F*_o_^2^ + 2*F*_c_^2^)/3
2939 reflections	(Δ/σ)_max_ = 0.001
333 parameters	Δρ_max_ = 0.14 e Å^−^^3^
0 restraints	Δρ_min_ = −0.21 e Å^−^^3^

### Special details

**Table:**

Experimental. — NMR spectra were recorded on a Varian INOVA-400 instrument in CDCl_3_ at 298 K. For ^1^H and ^13^C{^1^H} spectra, the TMS resonances (δ_H_ = 0.0 and δ_C_ = 0.0) were used as internal reference standards. — Chromato-mass spectrum was measured on Agilent 6890 Series GC system equipped with HP 5973 mass-selective detector. — ^1^H NMR: δ = 2.93 (m, 2 H, Indenyl—CH_2_), 3.34 (m, 2 H, CH_2_ in indene), 3.95 (m, 2 H, NCH_2_), 6.27 (m, 1 H, C═CH in indene), 7.15 (br s, 2 H, HC═CH in imidazole), 7.21, 7.30, 7.38, 7.46 (all m, all 1 H, CH in benzene ring of indene), 8.22 (m, 1 H, HC═N). — ^13^C{^1^H} NMR: δ = 29.02 (Indenyl—CH_2_), 37.78 (NCH_2_), 59.13 (CH_2_ in indene), 118.70 (═ CH in indene), 118.23, 130.60 (both br, HC═CH in imidazole), 123.79, 124.68, 125.98, 129.24 (CH in benzene ring of indene), 141.38 (═C in indene), 144.24, 144.88 (C in benzene ring of indene), 152.86 (HC═N). — EI MS (70 eV) m/*z* (%): 237 (8) [*M*], 141 (9) [benztropilium], 128 (28) [benzpentafulvene], 115 (13) [indenilium], 109 (100) [C_5_H_7_N_3_], 108 (36) [C_5_H_6_N_3_], 82 (25) [C_4_H_6_N_2_], 81 (82) [C_4_H_5_N_2_].
Geometry. All e.s.d.'s (except the e.s.d. in the dihedral angle between two l.s. planes) are estimated using the full covariance matrix. The cell e.s.d.'s are taken into account individually in the estimation of e.s.d.'s in distances, angles and torsion angles; correlations between e.s.d.'s in cell parameters are only used when they are defined by crystal symmetry. An approximate (isotropic) treatment of cell e.s.d.'s is used for estimating e.s.d.'s involving l.s. planes.
Refinement. Refinement of *F*^2^ against ALL reflections. The weighted *R*-factor *wR* and goodness of fit *S* are based on *F*^2^, conventional *R*-factors *R* are based on *F*, with *F* set to zero for negative *F*^2^. The threshold expression of *F*^2^ > σ(*F*^2^) is used only for calculating *R*-factors(gt) *etc*. and is not relevant to the choice of reflections for refinement. *R*-factors based on *F*^2^ are statistically about twice as large as those based on *F*, and *R*- factors based on ALL data will be even larger.

### Fractional atomic coordinates and isotropic or equivalent isotropic displacement parameters (Å^2^)

**Table:**

	*x*	*y*	*z*	*U*_iso_*/*U*_eq_	
N1A	0.6784 (5)	0.2550 (3)	0.62194 (4)	0.0481 (6)	
H1A	0.616 (7)	0.334 (5)	0.6273 (7)	0.092 (15)*	
N2A	0.9145 (4)	0.0506 (3)	0.61856 (4)	0.0490 (6)	
N3A	0.9748 (5)	0.3849 (3)	0.66146 (4)	0.0510 (6)	
C1A	0.8760 (5)	0.1896 (3)	0.63011 (5)	0.0439 (7)	
C2A	0.5871 (6)	0.1520 (4)	0.60450 (5)	0.0549 (8)	
H2A	0.4512	0.1645	0.5956	0.066*	
C3A	0.7330 (5)	0.0284 (4)	0.60277 (5)	0.0534 (8)	
H3A	0.7125	−0.0604	0.5922	0.064*	
C4A	1.0258 (5)	0.2616 (4)	0.64897 (5)	0.0478 (7)	
H4A	1.1664	0.2141	0.6519	0.057*	
C5A	1.1416 (6)	0.4457 (4)	0.67957 (5)	0.0556 (8)	
H5AA	1.1685	0.5587	0.6762	0.067*	
H5AB	1.2841	0.3889	0.6772	0.067*	
C6A	1.0601 (5)	0.4243 (4)	0.70698 (5)	0.0510 (8)	
H6AA	0.9112	0.4731	0.7086	0.061*	
H6AB	1.0437	0.3105	0.7104	0.061*	
C7A	1.2152 (5)	0.4959 (3)	0.72690 (5)	0.0407 (6)	
C8A	1.4034 (5)	0.5814 (4)	0.72351 (5)	0.0518 (7)	
H8A	1.4662	0.6050	0.7075	0.062*	
C9A	1.5019 (6)	0.6351 (4)	0.74873 (7)	0.0600 (8)	
H9AA	1.6560	0.5959	0.7509	0.072*	
H9AB	1.5016	0.7512	0.7501	0.072*	
C10A	1.3448 (5)	0.5610 (3)	0.76792 (5)	0.0463 (7)	
C11A	1.1729 (5)	0.4801 (3)	0.75462 (5)	0.0393 (6)	
C12A	1.0062 (5)	0.3973 (3)	0.76790 (5)	0.0500 (7)	
H12A	0.8936	0.3419	0.7590	0.060*	
C13A	1.0092 (7)	0.3980 (4)	0.79442 (5)	0.0621 (9)	
H13A	0.8967	0.3441	0.8036	0.074*	
C14A	1.1783 (7)	0.4783 (5)	0.80745 (6)	0.0680 (10)	
H14A	1.1796	0.4766	0.8254	0.082*	
C15A	1.3455 (7)	0.5612 (4)	0.79457 (6)	0.0654 (10)	
H15A	1.4572	0.6164	0.8036	0.078*	
N1B	0.2511 (4)	0.7865 (3)	0.62860 (4)	0.0468 (6)	
H1B	0.143 (6)	0.860 (4)	0.6239 (6)	0.071 (11)*	
N2B	0.4626 (4)	0.5689 (3)	0.62951 (4)	0.0510 (6)	
N3B	−0.0309 (5)	0.6341 (3)	0.59023 (4)	0.0513 (6)	
C1B	0.2802 (5)	0.6370 (4)	0.61924 (5)	0.0443 (7)	
C2B	0.4232 (5)	0.8149 (4)	0.64549 (5)	0.0541 (8)	
H2B	0.4474	0.9081	0.6549	0.065*	
C3B	0.5514 (5)	0.6812 (4)	0.64584 (5)	0.0527 (8)	
H3B	0.6816	0.6673	0.6557	0.063*	
C4B	0.1292 (5)	0.5614 (4)	0.60103 (5)	0.0473 (7)	
H4B	0.1518	0.4536	0.5971	0.057*	
C5B	−0.1762 (6)	0.5435 (4)	0.57281 (5)	0.0577 (8)	
H5BA	−0.3337	0.5575	0.5778	0.069*	
H5BB	−0.1399	0.4302	0.5741	0.069*	
C6B	−0.1461 (5)	0.5976 (4)	0.54526 (5)	0.0497 (7)	
H6BA	−0.1654	0.7131	0.5444	0.060*	
H6BB	0.0077	0.5731	0.5398	0.060*	
C7B	−0.3092 (5)	0.5204 (3)	0.52707 (5)	0.0414 (6)	
C8B	−0.4903 (5)	0.4293 (4)	0.53233 (5)	0.0529 (7)	
H8B	−0.5359	0.4018	0.5489	0.063*	
C9B	−0.6111 (5)	0.3769 (4)	0.50844 (6)	0.0560 (8)	
H9BA	−0.6134	0.2609	0.5070	0.067*	
H9BB	−0.7658	0.4170	0.5081	0.067*	
C10B	−0.4709 (5)	0.4507 (3)	0.48763 (5)	0.0459 (7)	
C11B	−0.2911 (4)	0.5363 (3)	0.49889 (5)	0.0388 (6)	
C12B	−0.1338 (5)	0.6134 (4)	0.48353 (5)	0.0507 (7)	
H12B	−0.0144	0.6706	0.4908	0.061*	
C13B	−0.1569 (6)	0.6040 (4)	0.45713 (6)	0.0631 (9)	
H13B	−0.0517	0.6558	0.4467	0.076*	
C14B	−0.3317 (6)	0.5201 (4)	0.44599 (6)	0.0642 (9)	
H14B	−0.3437	0.5154	0.4281	0.077*	
C15B	−0.4900 (6)	0.4426 (4)	0.46121 (6)	0.0578 (8)	
H15B	−0.6085	0.3854	0.4537	0.069*	

### Atomic displacement parameters (Å^2^)

**Table:**

	*U*^11^	*U*^22^	*U*^33^	*U*^12^	*U*^13^	*U*^23^
N1A	0.0537 (16)	0.0473 (15)	0.0431 (12)	0.0049 (13)	−0.0006 (12)	−0.0032 (11)
N2A	0.0555 (15)	0.0510 (14)	0.0405 (11)	0.0040 (13)	−0.0006 (11)	−0.0071 (11)
N3A	0.0610 (16)	0.0558 (15)	0.0361 (10)	0.0018 (14)	−0.0071 (12)	−0.0014 (11)
C1A	0.0502 (16)	0.0463 (16)	0.0352 (12)	0.0020 (14)	0.0010 (13)	0.0042 (12)
C2A	0.0556 (18)	0.060 (2)	0.0493 (15)	−0.0018 (17)	−0.0098 (15)	−0.0027 (14)
C3A	0.0610 (18)	0.0567 (19)	0.0425 (14)	−0.0020 (17)	−0.0021 (14)	−0.0092 (14)
C4A	0.0515 (17)	0.0516 (17)	0.0404 (13)	0.0030 (15)	−0.0016 (14)	0.0024 (13)
C5A	0.0615 (19)	0.057 (2)	0.0488 (15)	−0.0048 (17)	−0.0009 (15)	−0.0056 (14)
C6A	0.0495 (17)	0.060 (2)	0.0429 (13)	−0.0078 (16)	−0.0047 (13)	−0.0053 (13)
C7A	0.0378 (14)	0.0359 (14)	0.0485 (14)	0.0015 (13)	−0.0039 (12)	−0.0006 (12)
C8A	0.0460 (16)	0.0516 (18)	0.0578 (16)	−0.0050 (15)	−0.0026 (14)	0.0062 (14)
C9A	0.0421 (15)	0.0497 (17)	0.088 (2)	−0.0053 (15)	−0.0163 (16)	−0.0005 (16)
C10A	0.0431 (15)	0.0377 (15)	0.0582 (16)	0.0068 (14)	−0.0153 (14)	−0.0095 (13)
C11A	0.0414 (15)	0.0312 (13)	0.0453 (13)	0.0047 (12)	−0.0065 (12)	−0.0030 (11)
C12A	0.0539 (17)	0.0439 (16)	0.0521 (15)	−0.0039 (15)	−0.0037 (15)	−0.0002 (13)
C13A	0.076 (2)	0.062 (2)	0.0486 (15)	0.007 (2)	0.0017 (17)	0.0053 (15)
C14A	0.088 (3)	0.072 (2)	0.0447 (16)	0.022 (2)	−0.0061 (18)	−0.0082 (16)
C15A	0.073 (2)	0.060 (2)	0.0635 (19)	0.010 (2)	−0.0289 (18)	−0.0162 (17)
N1B	0.0498 (15)	0.0462 (14)	0.0445 (12)	0.0052 (13)	−0.0047 (12)	−0.0026 (11)
N2B	0.0510 (15)	0.0540 (15)	0.0480 (12)	0.0078 (13)	0.0008 (12)	0.0008 (12)
N3B	0.0612 (15)	0.0544 (15)	0.0384 (11)	−0.0022 (14)	−0.0042 (12)	−0.0049 (11)
C1B	0.0484 (16)	0.0462 (16)	0.0382 (12)	0.0013 (14)	0.0033 (13)	0.0007 (12)
C2B	0.0565 (19)	0.0560 (19)	0.0498 (15)	−0.0073 (17)	−0.0081 (15)	−0.0078 (14)
C3B	0.0495 (17)	0.062 (2)	0.0464 (15)	−0.0006 (16)	−0.0071 (14)	0.0015 (14)
C4B	0.0630 (18)	0.0413 (15)	0.0376 (13)	0.0011 (15)	0.0012 (13)	0.0026 (12)
C5B	0.0613 (19)	0.063 (2)	0.0486 (15)	−0.0129 (18)	−0.0072 (15)	−0.0007 (14)
C6B	0.0500 (17)	0.0567 (18)	0.0422 (13)	−0.0045 (15)	−0.0042 (13)	−0.0030 (13)
C7B	0.0404 (14)	0.0396 (15)	0.0442 (13)	−0.0017 (13)	−0.0025 (12)	−0.0034 (12)
C8B	0.0511 (17)	0.0542 (18)	0.0532 (15)	−0.0044 (16)	0.0016 (14)	0.0039 (14)
C9B	0.0413 (16)	0.0535 (18)	0.0732 (19)	−0.0068 (15)	−0.0060 (15)	−0.0093 (15)
C10B	0.0419 (15)	0.0378 (14)	0.0580 (15)	0.0063 (13)	−0.0094 (14)	−0.0076 (13)
C11B	0.0378 (13)	0.0297 (13)	0.0489 (14)	0.0034 (12)	−0.0062 (12)	−0.0042 (11)
C12B	0.0501 (17)	0.0502 (17)	0.0520 (15)	−0.0043 (16)	−0.0040 (14)	−0.0018 (13)
C13B	0.067 (2)	0.071 (2)	0.0509 (16)	0.000 (2)	0.0056 (16)	−0.0003 (16)
C14B	0.072 (2)	0.074 (2)	0.0472 (15)	0.014 (2)	−0.0115 (17)	−0.0101 (16)
C15B	0.0548 (18)	0.0578 (19)	0.0608 (17)	0.0079 (17)	−0.0197 (16)	−0.0173 (15)

### Geometric parameters (Å, °)

**Table:**

N1A—C1A	1.352 (4)	N1B—C1B	1.348 (4)
N1A—C2A	1.358 (4)	N1B—C2B	1.360 (4)
N1A—H1A	0.80 (4)	N1B—H1B	0.92 (4)
N2A—C1A	1.324 (4)	N2B—C1B	1.326 (4)
N2A—C3A	1.359 (4)	N2B—C3B	1.366 (4)
N3A—C4A	1.251 (3)	N3B—C4B	1.253 (4)
N3A—C5A	1.451 (4)	N3B—C5B	1.456 (4)
C1A—C4A	1.448 (4)	C1B—C4B	1.442 (4)
C2A—C3A	1.344 (4)	C2B—C3B	1.345 (4)
C2A—H2A	0.9300	C2B—H2B	0.9300
C3A—H3A	0.9300	C3B—H3B	0.9300
C4A—H4A	0.9300	C4B—H4B	0.9300
C5A—C6A	1.512 (4)	C5B—C6B	1.510 (4)
C5A—H5AA	0.9700	C5B—H5BA	0.9700
C5A—H5AB	0.9700	C5B—H5BB	0.9700
C6A—C7A	1.502 (4)	C6B—C7B	1.492 (4)
C6A—H6AA	0.9700	C6B—H6BA	0.9700
C6A—H6AB	0.9700	C6B—H6BB	0.9700
C7A—C8A	1.328 (4)	C7B—C8B	1.336 (4)
C7A—C11A	1.466 (3)	C7B—C11B	1.473 (3)
C8A—C9A	1.500 (4)	C8B—C9B	1.495 (4)
C8A—H8A	0.9300	C8B—H8B	0.9300
C9A—C10A	1.492 (5)	C9B—C10B	1.491 (4)
C9A—H9AA	0.9700	C9B—H9BA	0.9700
C9A—H9AB	0.9700	C9B—H9BB	0.9700
C10A—C15A	1.383 (4)	C10B—C15B	1.377 (4)
C10A—C11A	1.397 (4)	C10B—C11B	1.403 (4)
C11A—C12A	1.383 (4)	C11B—C12B	1.380 (4)
C12A—C13A	1.377 (4)	C12B—C13B	1.379 (4)
C12A—H12A	0.9300	C12B—H12B	0.9300
C13A—C14A	1.377 (5)	C13B—C14B	1.371 (5)
C13A—H13A	0.9300	C13B—H13B	0.9300
C14A—C15A	1.375 (5)	C14B—C15B	1.382 (5)
C14A—H14A	0.9300	C14B—H14B	0.9300
C15A—H15A	0.9300	C15B—H15B	0.9300
			
C1A—N1A—C2A	107.1 (3)	C1B—N1B—C2B	107.4 (3)
C1A—N1A—H1A	128 (3)	C1B—N1B—H1B	128 (2)
C2A—N1A—H1A	124 (3)	C2B—N1B—H1B	125 (2)
C1A—N2A—C3A	104.9 (3)	C1B—N2B—C3B	105.4 (3)
C4A—N3A—C5A	117.4 (3)	C4B—N3B—C5B	118.0 (3)
N2A—C1A—N1A	111.0 (3)	N2B—C1B—N1B	110.6 (3)
N2A—C1A—C4A	124.4 (3)	N2B—C1B—C4B	125.1 (3)
N1A—C1A—C4A	124.7 (3)	N1B—C1B—C4B	124.2 (3)
C3A—C2A—N1A	106.0 (3)	C3B—C2B—N1B	106.4 (3)
C3A—C2A—H2A	127.0	C3B—C2B—H2B	126.8
N1A—C2A—H2A	127.0	N1B—C2B—H2B	126.8
C2A—C3A—N2A	110.9 (3)	C2B—C3B—N2B	110.1 (3)
C2A—C3A—H3A	124.5	C2B—C3B—H3B	124.9
N2A—C3A—H3A	124.5	N2B—C3B—H3B	124.9
N3A—C4A—C1A	123.0 (3)	N3B—C4B—C1B	123.0 (3)
N3A—C4A—H4A	118.5	N3B—C4B—H4B	118.5
C1A—C4A—H4A	118.5	C1B—C4B—H4B	118.5
N3A—C5A—C6A	110.7 (2)	N3B—C5B—C6B	111.4 (3)
N3A—C5A—H5AA	109.5	N3B—C5B—H5BA	109.4
C6A—C5A—H5AA	109.5	C6B—C5B—H5BA	109.4
N3A—C5A—H5AB	109.5	N3B—C5B—H5BB	109.4
C6A—C5A—H5AB	109.5	C6B—C5B—H5BB	109.4
H5AA—C5A—H5AB	108.1	H5BA—C5B—H5BB	108.0
C7A—C6A—C5A	114.1 (2)	C7B—C6B—C5B	113.3 (3)
C7A—C6A—H6AA	108.7	C7B—C6B—H6BA	108.9
C5A—C6A—H6AA	108.7	C5B—C6B—H6BA	108.9
C7A—C6A—H6AB	108.7	C7B—C6B—H6BB	108.9
C5A—C6A—H6AB	108.7	C5B—C6B—H6BB	108.9
H6AA—C6A—H6AB	107.6	H6BA—C6B—H6BB	107.7
C8A—C7A—C11A	108.6 (3)	C8B—C7B—C11B	108.2 (2)
C8A—C7A—C6A	128.9 (3)	C8B—C7B—C6B	128.9 (2)
C11A—C7A—C6A	122.5 (2)	C11B—C7B—C6B	122.9 (2)
C7A—C8A—C9A	111.5 (3)	C7B—C8B—C9B	112.0 (3)
C7A—C8A—H8A	124.3	C7B—C8B—H8B	124.0
C9A—C8A—H8A	124.3	C9B—C8B—H8B	124.0
C10A—C9A—C8A	102.7 (3)	C10B—C9B—C8B	102.6 (2)
C10A—C9A—H9AA	111.2	C10B—C9B—H9BA	111.2
C8A—C9A—H9AA	111.2	C8B—C9B—H9BA	111.2
C10A—C9A—H9AB	111.2	C10B—C9B—H9BB	111.2
C8A—C9A—H9AB	111.2	C8B—C9B—H9BB	111.2
H9AA—C9A—H9AB	109.1	H9BA—C9B—H9BB	109.2
C15A—C10A—C11A	119.8 (3)	C15B—C10B—C11B	120.1 (3)
C15A—C10A—C9A	131.7 (3)	C15B—C10B—C9B	131.0 (3)
C11A—C10A—C9A	108.5 (2)	C11B—C10B—C9B	108.9 (2)
C12A—C11A—C10A	120.5 (2)	C12B—C11B—C10B	120.1 (2)
C12A—C11A—C7A	130.8 (3)	C12B—C11B—C7B	131.6 (3)
C10A—C11A—C7A	108.6 (2)	C10B—C11B—C7B	108.3 (2)
C13A—C12A—C11A	119.2 (3)	C13B—C12B—C11B	118.8 (3)
C13A—C12A—H12A	120.4	C13B—C12B—H12B	120.6
C11A—C12A—H12A	120.4	C11B—C12B—H12B	120.6
C14A—C13A—C12A	120.2 (3)	C14B—C13B—C12B	121.5 (3)
C14A—C13A—H13A	119.9	C14B—C13B—H13B	119.3
C12A—C13A—H13A	119.9	C12B—C13B—H13B	119.3
C15A—C14A—C13A	121.5 (3)	C13B—C14B—C15B	120.2 (3)
C15A—C14A—H14A	119.3	C13B—C14B—H14B	119.9
C13A—C14A—H14A	119.3	C15B—C14B—H14B	119.9
C14A—C15A—C10A	118.9 (3)	C10B—C15B—C14B	119.4 (3)
C14A—C15A—H15A	120.5	C10B—C15B—H15B	120.3
C10A—C15A—H15A	120.5	C14B—C15B—H15B	120.3
			
C3A—N2A—C1A—N1A	−0.4 (3)	C3B—N2B—C1B—N1B	−0.4 (3)
C3A—N2A—C1A—C4A	179.5 (3)	C3B—N2B—C1B—C4B	−178.9 (3)
C2A—N1A—C1A—N2A	0.3 (3)	C2B—N1B—C1B—N2B	0.2 (3)
C2A—N1A—C1A—C4A	−179.6 (3)	C2B—N1B—C1B—C4B	178.8 (3)
C1A—N1A—C2A—C3A	−0.1 (3)	C1B—N1B—C2B—C3B	0.0 (3)
N1A—C2A—C3A—N2A	−0.2 (3)	N1B—C2B—C3B—N2B	−0.2 (3)
C1A—N2A—C3A—C2A	0.4 (3)	C1B—N2B—C3B—C2B	0.4 (3)
C5A—N3A—C4A—C1A	−179.3 (2)	C5B—N3B—C4B—C1B	−178.1 (3)
N2A—C1A—C4A—N3A	−172.7 (3)	N2B—C1B—C4B—N3B	−174.1 (3)
N1A—C1A—C4A—N3A	7.3 (4)	N1B—C1B—C4B—N3B	7.5 (4)
C4A—N3A—C5A—C6A	−111.5 (3)	C4B—N3B—C5B—C6B	−112.1 (3)
N3A—C5A—C6A—C7A	−175.5 (3)	N3B—C5B—C6B—C7B	−173.8 (3)
C5A—C6A—C7A—C8A	4.2 (5)	C5B—C6B—C7B—C8B	9.4 (5)
C5A—C6A—C7A—C11A	−176.5 (3)	C5B—C6B—C7B—C11B	−170.9 (3)
C11A—C7A—C8A—C9A	−1.6 (3)	C11B—C7B—C8B—C9B	−0.4 (3)
C6A—C7A—C8A—C9A	177.8 (3)	C6B—C7B—C8B—C9B	179.3 (3)
C7A—C8A—C9A—C10A	2.2 (3)	C7B—C8B—C9B—C10B	0.5 (3)
C8A—C9A—C10A—C15A	178.0 (3)	C8B—C9B—C10B—C15B	177.8 (3)
C8A—C9A—C10A—C11A	−2.0 (3)	C8B—C9B—C10B—C11B	−0.3 (3)
C15A—C10A—C11A—C12A	−1.4 (4)	C15B—C10B—C11B—C12B	0.4 (4)
C9A—C10A—C11A—C12A	178.6 (3)	C9B—C10B—C11B—C12B	178.8 (3)
C15A—C10A—C11A—C7A	−178.8 (3)	C15B—C10B—C11B—C7B	−178.3 (3)
C9A—C10A—C11A—C7A	1.2 (3)	C9B—C10B—C11B—C7B	0.1 (3)
C8A—C7A—C11A—C12A	−176.8 (3)	C8B—C7B—C11B—C12B	−178.2 (3)
C6A—C7A—C11A—C12A	3.8 (5)	C6B—C7B—C11B—C12B	2.0 (5)
C8A—C7A—C11A—C10A	0.2 (3)	C8B—C7B—C11B—C10B	0.2 (3)
C6A—C7A—C11A—C10A	−179.2 (3)	C6B—C7B—C11B—C10B	−179.6 (3)
C10A—C11A—C12A—C13A	1.2 (4)	C10B—C11B—C12B—C13B	−0.1 (4)
C7A—C11A—C12A—C13A	177.9 (3)	C7B—C11B—C12B—C13B	178.1 (3)
C11A—C12A—C13A—C14A	−0.9 (5)	C11B—C12B—C13B—C14B	−0.1 (5)
C12A—C13A—C14A—C15A	0.9 (5)	C12B—C13B—C14B—C15B	0.1 (5)
C13A—C14A—C15A—C10A	−1.1 (5)	C11B—C10B—C15B—C14B	−0.4 (4)
C11A—C10A—C15A—C14A	1.3 (5)	C9B—C10B—C15B—C14B	−178.3 (3)
C9A—C10A—C15A—C14A	−178.7 (3)	C13B—C14B—C15B—C10B	0.1 (5)

### Hydrogen-bond geometry (Å, °)

**Table:**

*D*—H···*A*	*D*—H	H···*A*	*D*···*A*	*D*—H···*A*
N1A—H1A···N2B	0.80 (4)	2.16 (4)	2.935 (4)	162 (4)
N1B—H1B···N2A^i^	0.92 (4)	2.10 (4)	3.006 (4)	170 (3)

Symmetry codes: (i) *x*−1, *y*+1, *z*.
